# Do Early Diagnosis and Glucocorticoid Treatment Decrease the Risk of Permanent Visual Loss and Early Relapses in Giant Cell Arteritis: A Prospective Longitudinal Study

**DOI:** 10.1097/MD.0000000000003210

**Published:** 2016-04-08

**Authors:** Alojzija Hocevar, Ziga Rotar, Rok Jese, Snezna Sodin Semrl, Joze Pizem, Marko Hawlina, Matija Tomsic

**Affiliations:** From the Department of Rheumatology (AH, ZR, RJ, SSS, MT); Department of Ophthalmology (MH); University Medical Centre Ljubljana, Institute of Pathology (JP); Faculty of Medicine (MT), University of Ljubljana, Ljubljana, Slovenia; and Faculty of Mathematics, Natural Science and Information Technology (SSS), University of Primorska, Koper, Slovenia.

## Abstract

To determine the incidence of permanent visual loss (PVL) in giant cell arteritis (GCA) and the GCA relapse rate during glucocorticoid (GC) tapering.

This prospective, longitudinal single secondary/tertiary rheumatology centre study was conducted between September 2011 and September 2014 in Slovenia. Predetermined clinical and laboratory tests were performed at 12, 24, 48, 96, and 144 weeks after diagnosis.

Sixty-eight GCA patients (72.1% female), with a median (IQR) age of 73.2 (67.3–76.1) years and a symptom duration before the diagnosis of a median (IQR) 30 (14–70) days were included. Thirty-nine of 68 patients had symptoms for less than 31 days (14 (10–28) days–early GCA) and 29/68 for 31 days or longer (90 (60–120) days–late GCA). Four (5.9%) patients presented with PVL (1 early GCA). The median (IQR) follow-up was (IQR) 104 (53–126) weeks. GCA relapsed in 17/39 (43.6%) and 14/29 (48.3%) in early and late GCA, respectively. The median (IQR) time to the first relapse was 24.8 (13.6–46.5) weeks (early GCA 14 (13–34) weeks; late GCA 25 (22–48) weeks, *P* = 0.117), at the methyl-prednisolone dose of 6.0 (4.0–12.0) mg. The patients who relapsed had significantly higher levels of inflammation parameters at the baseline (including ESR, CRP, serum amyloid A, haptoglobin, and fibrinogen).

An early GCA diagnosis and prompt GC treatment decreased the PVL rate in comparison to historic controls, but seem to have no impact on the frequency of relapses, which are predicted by the high baseline levels of the biomarkers of inflammation.

## INTRODUCTION

Giant cell arteritis (GCA) is a systemic vasculitis affecting the large and medium-sized arteries.^1^ It is the most common primary vasculitis in adults over the age of 50 years, with an annual incidence rate of 1.6 to 32.8 cases per 10^5^ persons in this age group.^2,3^ Despite our improved knowledge, GCA retains a high morbidity burden, with the risk of early, severe ischemic complications, and increasingly recognized “large vessel” disease. The disease often takes a chronic course with frequent flares and a need for prolonged immunosuppressive treatment and consequent treatment-related adverse events.

The permanent visual loss (PVL) affected 15% to 20% of patients in historical cohorts and represents one of the most devastating complications of GCA.^4,5^ The anterior ischemic optic neuropathy and central retinal artery occlusion are the most common causes of PVL, which may be prevented by an early intervention.^6^

Two recent studies showed early relapses during treatment with glucocorticoids (GC). The relapses most commonly occurred within the first 2 years of treatment.^7^ Nearly 75% of relapses in newly diagnosed GCA cases occurred at a median prednisone dose of 10 mg qd.^8^ Predictors of relapses are poorly understood. It had been suggested that patients with large vessel GCA (lvGCA), a strong initial systemic inflammatory response, and a hemoglobin level below 120 mg/l at the baseline, predict a higher risk of a relapse.^9–12^

We performed a prospective, longitudinal observational study to determine the incidence of PVL in GCA with respect to the promptness of the diagnosis and the initiation of GC treatment, the GCA relapse rate during GC tapering, and the potential predictors of a GCA relapse.

## METHODS

### Setting

This prospective, longitudinal observational study was performed at the Department of Rheumatology, University Medical Centre Ljubljana—an integrated teaching hospital, serving approximately 1,060,000 adult residents on the tertiary level, and is the only secondary level referral hospital in the Ljubljana region, serving approximately 530,000 adult residents, so most of the patients suspected of having GCA are referred to our early interventional clinic.

### Patients and Diagnosis

New cases of cranial GCA (cGCA) and lvGCA diagnosed between September 2011 and September 2014 which were prospectively followed for at least 48 weeks were included in the final analysis.

The diagnosis of cGCA was based on the 1990 ACR classification criteria, and a positive temporal artery biopsy (TAB) or the presence of the “halo” sign on temporal artery color Doppler sonography (CDS). In cases of suspected lvGCA CDS of the large epiaortic arteries (carotid, vertebral, subclavian, axillary, and brachial arteries) or positron emission tomography/computed tomography (PET/CT) or both were performed.

### Baseline and Follow-Up Evaluation

Baseline patient work-up included a meticulous history of the symptoms of GCA, comorbidities, a thorough physical examination, extensive laboratory and imaging tests and, in most cases, TAB.

Follow-up visits with predetermined clinical and laboratory tests were performed at 12, 24, 48, 96, and 144 weeks after diagnosis by 2 rheumatologists (AH or RJ). Additional unscheduled visits were arranged for patients who relapsed during the GC tapering.

The parameters of inflammation routinely followed included erythrocyte sedimentation rate (ESR), C-reactive protein (CRP), ferritin, haptoglobin, fibrinogen, serum amyloid A (SAA), and interleukin-6 (IL-6).

### Treatment

Treatment was initiated at the time of diagnosis in all patients and followed the unified protocol in line with the EULAR recommendations.^13^ Patients with uncomplicated cGCA were initially treated with oral methyl-prednisolone (MP) 32 to 48 mg qd, while those with ischemic complications (visual disturbances, stroke) or those with lvGCA first received MP 250 mg on 3 consecutive days intravenously. MP tapering was started 2 to 4 weeks after treatment initiation, slowly reducing the dose by 4 mg every week until reaching an MP dose of 16 mg. Afterwards the MP was reduced by 2 mg every other week. When reaching 8 mg MP, the dose was maintained for 1 month. Thereafter the MP was reduced by 1 mg every month, until reaching 4 mg qd, which was then continued for at least 1.5 years.

All patients were treated, unless contraindicated, with acetylsalicylic acid 100 mg. At a relapse the dose of MP was increased. Leflunomide (20 mg qd) or methotrexate (starting dose 15 mg qwk, titrating up to 20 mg qwk) was used as glucocorticoid sparing agents. Leflunomide was the initial choice, and methotrexate was prescribed in case of leflunomide associated significant adverse events or to attain better treatment adherence.

### Stratification of the Cohort and the Definition of Terms

For the purposes of analysis we stratified the patients by disease type (cGCA, lvGCA), by symptom duration to diagnosis and initiation of treatment (early GCA if diagnosed and treated within 30 days of the symptom onset or late GCA if diagnosed at a later stage) and by their relapse status (relapsed, nonrelapsed).

*PVL* was defined as a new onset of a permanent reduction of visual acuity or visual field loss on the affected eye.

*Disease relapse* was defined as disease worsening or new disease activity during GC tapering after the initial remission. Other reasons for the disease-related symptoms (ie, infections, malignancy) had to be excluded.

### Statistical Analysis

MedCalc Version 12.2 was used for the statistical analyses. The Mann–Whitney test was used to test the differences among subgroups of patients for metric variables. The Fisher exact test was used in case of categorical variables. The Kaplan–Meier method was used to evaluate the time to the first relapse.

### Ethics Committee Approval

The study was approved by the Slovenian National Medical Ethics Committee.

## RESULTS

### Patient Demographics, Clinical Presentation, Diagnostic Work-Up at the Baseline and Initial Treatment

During the observation period of 48 months, 73 new GCA cases were identified. Two patients died (1 of cancer during the initial diagnostic work-up, 1 due to causes unrelated to vasculitis after 6 months of follow-up). One patient refused the treatment and 2 were lost to follow-up. The baseline characteristics of the remaining 68 are presented in Table 1, column A.

**TABLE 1:**
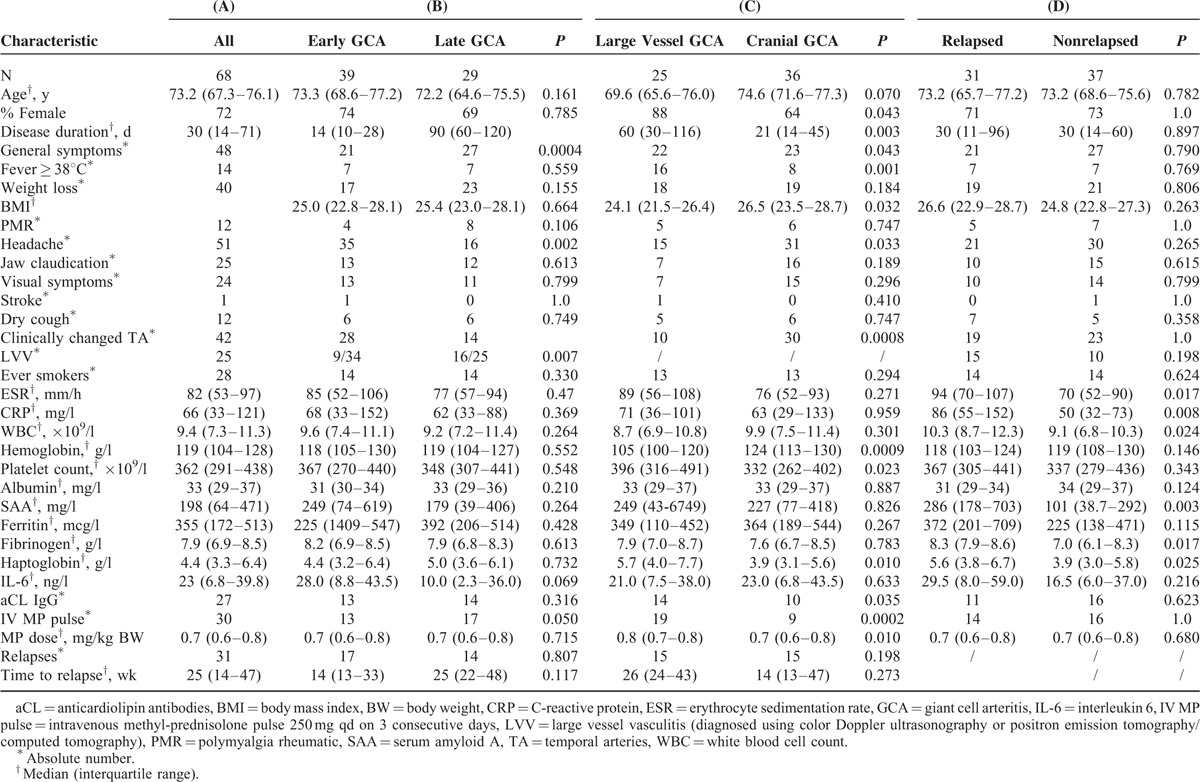
Patient Characteristics

The diagnostic work-up was completed in median 1 day from the presentation at our early interventional clinic. The median (IQR) time to the temporal artery CDS was 0 (0–0) days and to the TAB 1 (0–1) day, with the preliminary histological analysis results available in 3 hours. The TAB was performed in 55/68 (81%) cases and was positive in 45/55 (82%) cases. The CDS of the temporal arteries was performed in 67/68 (99%) patients. The “halo” sign was found in 52/67 (78%; 40% unilateral, 60% bilateral). A CDS of the large epiaortic arteries was performed in 61/68 (90%) patients and was consistent with lvGCA in 22/61 (36%). PET/CT was done in 11/68 (16%) patients, and was consistent with lvGCA in all cases (including 3 patients with normal epiaortic vessels on CDS).

Overall 59/68 (87%) patients fulfilled the ACR classification criteria, including 16/25 (64%) patients with lvGCA. In the remaining nine patients with lvGCA, the diagnosis was based on the result of the CDS of large epiaortic arteries (2 patients), PET/CT (1 patient), or both (6 patients).

Thirty patients (44%) were initially treated with an intravenous MP pulse. The mean (SD) initial dose of oral MP was 47 (5) mg or 0.7 mg/kg body weight. At the time of diagnosis, 17 patients had already been taking acetylsalicylic acid and 6 were on anticoagulant therapy. In all the others (45/68), acetylsalicylic acid was prescribed for the first time concomitantly with glucocorticoids.

Baseline differences in the subgroups of patients with early versus late, lvGCA versus cCGA, and relapsed versus non-relapsed GC are shown in Table 1, columns B–D, respectively.

### Permanent Visual Loss

Four (5.9%) patients presented with a new onset of one sided PVL. Of the rest of the 35% of patients who presented with visual disturbances, 15 had blurred vision, 8 diplopia, 3 transient visual loss, and 1 ptosis. PVL was more common in lvGCA versus cGCA (3/25 vs 1/43, relative risk (RR) 5.7, *P* = 0.102) and in late versus early GCA (3/29 vs 1/39, RR 4.0, *P* = 0.177).

### Follow-Up and Predictors of a Relapse

**FIGURE 1:**
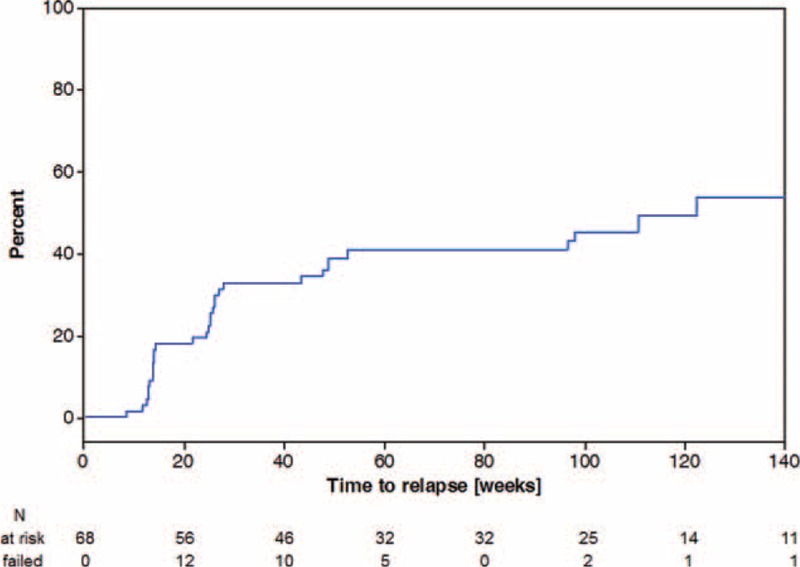
Overall cumulative failure probability plot (Kaplan–Meier method).

Sixty-eight GCA patients were followed for a median (IQR) 104 (53–126) weeks. During the observation period GCA relapsed in 31/68 (46%) of cases. Two GCA patients had 2 relapses, the remainder a single episode each. A cumulative failure probability plot is represented in Figure 1. The median (IQR) time to the first relapse was 24.8 (13.6–46.5) weeks at a median (IQR) MP dose of 6 (4–12) mg. A headache (15%) and general symptoms (39%) were the most common presentations of the relapse, followed by visual symptoms (13%; 1 case of PVL due to anterior ischemic optic neuropathy), jaw claudication (13%), symptoms of *polymyalgia rheumatica* (2%). Worsening of large vessel involvement (5%) was suggested in one patient with intermittent claudication of the hand that, in addition to medical treatment, required a percutaneous transluminal angioplasty with stenting of the axillary artery. In another patient surgical intervention was needed, due to the progression of the thoracic aortic aneurysm. In two additional patients, CDS of the large vessels implied worsening of the disease. In 29/31 (94%) of the relapsed patients an increase of inflammatory parameters was recorded at the time of the clinical relapse compared to the levels at remission. At relapse, the median (IQR, range) ESR was 40 (25–61, 11–78) mm/h and the CRP was 21 (13–39, 6–159) mg/l.

There were no significant differences in the clinical characteristics, including the presence of large vessel disease or time of the diagnosis between those who relapsed and those who remained in remission. However, the patients who relapsed had a significantly higher ESR, and CRP, SAA, haptoglobin, fibrinogen levels as well as white blood cell count at the baseline than those who did not (Table 1, column D).

There was no difference in the relapse rates between patients with early and late GCA (17/39 vs 14/29, *P* = 0.807), nor between those with cGCA and lvGCA (15/43 vs 15/25, *P* = 0.198). The differences in relapse rates, time to relapse among the subgroups are shown in the bottom two rows of Table 1, columns B–D.

In case of a relapse, the dosage of MP was temporary increased by 8 to 12 mg qd on top of the last previously effective dose. Glucocorticoid sparing therapy with leflunomide 20 mg qd, or methotrexate 15 mg qwk was started in 18/31 and 2/31 patients, respectively.

## DISCUSSION

During the past 2 decades it has become apparent that early diagnosis, prompt initiation of treatment, and frequent monitoring, improve the outcomes in certain chronic rheumatic diseases. In the case of GCA, the CDS and PET/CT have significantly aided early diagnosis and new treatment options for GCA are on the horizon. Yet the question remains, whether early diagnosis and early GC treatment represent a “window of opportunity” akin to that in, for example, rheumatoid arthritis? Two of the 3 recent studies showed that despite the availability of better diagnostic tools, the disease remained undiagnosed on average for 2 to 4 months which translates into a 2- to 4-month delay of treatment. Presently, no data are available on what the prognosis of GCA would be if it were diagnosed sooner.^10,12^

During the past 4 years we prospectively enrolled and followed patients to establish, to the best of our knowledge, a cohort of GCA patients with the shortest time to diagnosis in the available literature. The median duration of symptoms at the time of diagnosis and initiation of treatment was 30 days (≤30 days in 57%—early GCA). Early diagnosis and initiation of treatment are possible since rheumatologists perform the CDS and TAB themselves within a day of the first visit in our early interventional clinic and a preliminary histopathology report is obtainable within three hours of the TAB. We present the data on the incidence of PVL, the relapse rate and potential predictors of the relapse in this unique cohort considering time to diagnosis, and the subtype of GCA.

Our findings suggest that early diagnosis and treatment may save eyesight. Although ∼35% of patients complained of visual disturbances, less than 6% of our patients sustained a new onset of PVL at presentation compared to 15% to 20% previously reported. This observation is also implied within our own cohort where the incidence of PVL was higher in patients with lvGCA with whom it took us longer to establish the diagnosis, as well as with those with late GCA; although the differences were not statistically significant.

The window of opportunity for long term modification of the pathogenic immunologic response may be exceedingly short in GCA, or GC alone may be inadequate to induce a long-term remission. The relapse rate of ∼45% at median 25 weeks in our cohort is comparable with the above-mentioned follow-up studies. There was no difference in the relapse rate between patients with early and late GCA, or between those with cGCA or lvGCA. The latter finding is in contrast to the recent large cohort study which suggested earlier and more frequent relapses in lvGCA.^9^

Predictors of a GCA relapse have been addressed in a small number of studies. The comparison to the results with other studies is hindered by the fact that many studies did not stick to the strict definition of relapse and commonly reported pooled results on flares—that is, both relapses (flare while on GC treatment) and recurrences (flare after GC discontinuation). It had been shown that patients with a strong initial systemic inflammatory response have a prolonged disease course with more flares.^10,11^ Yet an intense inflammatory response at presentation signifies a lower risk of ischemic complications, while a prolonged intense inflammatory response predicts an increased risk of a relapse.^10^ Similarly, patients in our cohort who relapsed had a significantly higher ESR, CRP, SAA, haptoglobin, and fibrinogen levels at the baseline. However, we found no significant differences in the presenting clinical characteristics, including general symptoms (fever > 38°C, weight loss, fatigue), between those who relapsed and those who did not. Contrary to the Martinez-Lado et al paper,^12^ the difference in hemoglobin level at the baseline was not a significant predictor of a relapse in our cohort.

The most obvious limitations of our study are the size of the cohort, and the fact that it was performed at a single centre. We are aware of the potential selection bias since we did not include all the patients diagnosed and treated at other departments of the University Medical Centre Ljubljana. During the study period there were 9 additional cases of GCA at the department of Ophthalmology and three at the department of Infectious diseases of which 5 suffered PVL. These patients were not included since the available clinical, laboratory, and imaging data did not meet the rigorous diagnostic requirements of this study.

One might also question our ad hoc definition of early GCA due to the lack of a generally accepted definition.

The most compelling strong point of our study is certainly the prospectively diagnosed and followed GCA cohort that, with a thus far unmatched shortest lag between GCA symptom onset and diagnosis. Our cohort does not, with the exception of the early intervention and lower incidence of PVL, differ considerably from the historic cohorts, which further supports the credibility of our findings.^5^ We also believe that a high level of data consistency was maintained during the entire timespan of the study, since only 2 rheumatologists (AH, RJ) were involved in the evaluation and management of patients.

In conclusion, early diagnosis and prompt initiation of GC are conducive to a reduction in the incidence of PVL in GCA. However, even early diagnosis and a prompt initiation of GC do not prevent relapses. This may indicate that GC alone is inadequate to induce and sustain remission in GCA, in at least a considerable number of patients.
